# Postoperative platelet dynamic trajectories in patients with type A aortic dissection and development of a predictive model for thrombocytopenia

**DOI:** 10.3389/fcvm.2026.1948231

**Published:** 2026-10-07

**Authors:** Zeqi Bi, Yingliang Zhao, Chong Sheng, Yuanxi Luo, Yi Jiang, Dongjin Wang, Yunxing Xue

**Affiliations:** 1 Department of Cardiovascular Surgery, Nanjing Drum Tower Hospital, Peking Union Medical College Graduate School, Chinese Academy of Medical Sciences & Peking Union Medical College, Nanjing, China; 2 Department of Cardiovascular Surgery, Nanjing Drum Tower Hospital, Affiliated Hospital of Medical School, Nanjing University, Nanjing, China; 3 Institute of Cardiothoracic Vascular Disease, Nanjing University, Nanjing, China

**Keywords:** growth mixture model, platelet count, platelet dynamic trajectory, Stanford type A aortic dissection, thrombocytopenia

## Abstract

**Background:**

Thrombocytopenia is common after cardiac surgery and is associated with adverse postoperative outcomes. However, platelet counts are typically assessed at single time points, and longitudinal platelet trajectories after acute type A aortic dissection (ATAAD) surgery remain poorly characterized. This study used trajectory analysis to identify perioperative platelet count trajectories in patients undergoing ATAAD surgery.

**Methods:**

We retrospectively included 773 patients who underwent surgery for Stanford type A aortic dissection in this study. Platelet count data were collected over time within the first seven days after surgery. Trajectory patterns of platelet counts were determined using growth mixture modeling (GMM). The Kaplan–Meier method and log-rank test were applied to compare intergroup survival differences, and Cox regression analysis was performed to estimate mortality risks and stratify patient phenotypes. With trajectory phenotype as the outcome, least absolute shrinkage and selection operator (LASSO) regression was used for variable selection, followed by the construction of a logistic regression predictive model.

**Results:**

GMM identified three distinct nonlinear postoperative platelet trajectories, designated as TrajeA, TrajeB, and TrajeC. Kaplan–Meier survival analysis demonstrated significant differences in both early (postoperative days 7–30) and long-term survival among trajectory groups (log-rank *P* *<* *0.001*). TrajeC was associated with the highest mortality risk. Five clinical variables, namely age, preoperative creatinine, total transfusion volume, end-stage renal disease, and operative duration, were ultimately incorporated into the final logistic regression model. The model achieved an AUC of 0.73 (95% CI: 0.69–0.78) in the training cohort and 0.69 (95% CI: 0.61–0.76) in the validation cohort, demonstrating moderate, stable discriminative performance and no apparent overfitting.

**Conclusions:**

A persistently low postoperative platelet trajectory is associated with elevated short- and long-term mortality in patients undergoing acute type A aortic dissection surgery.

## Introduction

Acute type A aortic dissection (ATAAD) is a life-threatening cardiovascular emergency characterized by separation of the layers of the aortic wall. It usually triggers sudden, severe pain, and is immediately life-threatening because it can cause the aorta to rupture, leading to acute perfusion insufficiency in various organs (1, 2). Surgical repair remains the only definitive treatment for ATAAD (3). Therefore, early identification of patients at high risk for postoperative mortality and individualized intervention strategies are clinically crucial to improving survival in these patients.

Thrombocytopenia (platelet count <150 × 10⁹/L) (4, 5) is associated with increased morbidity and mortality (6, 7), as well as higher risks of bleeding, transfusion requirements, and the need for organ support (2). Beyond their role in hemostasis, platelets regulate inflammation, leukocyte interactions, and microvascular perfusion (6, 8, 9). In addition, infection is a common cause of thrombocytopenia; as one of the most frequent postoperative complications, infection can accelerate platelet consumption and impair platelet production, and the resulting thrombocytopenia in turn signals greater illness severity and an increased risk of adverse outcomes (10). In patients with ATAAD, thrombocytopenia complicates the clinical course in 8%–56% of ICU admissions (5). Tsilimigras et al. (11) demonstrated that patients undergoing major vascular surgery, particularly those with ATAAD, exhibit markedly accelerated platelet consumption secondary to aortic intimal laceration, collagen exposure, systemic inflammatory response, and activation of the coagulation cascade. Together with blood-extracorporeal circuit interactions, platelet activation and degranulation, and hypothermic injury from cardiopulmonary bypass or deep hypothermic circulatory arrest, these mechanisms contribute to a high incidence of severe postoperative thrombocytopenia (12). Subsequent studies have identified postoperative thrombocytopenia as an independent predictor of in-hospital mortality and reduced short-term survival following cardiac surgery (13). Bedel and Selvi (14) further reported that the platelet-to-lymphocyte ratio and neutrophil-to-lymphocyte ratio were significantly associated with in-hospital mortality in patients with ATAAD. However, these findings derive primarily from studies with small single-center cohorts or analyses restricted to single static postoperative measurements. In-depth characterization of longitudinal platelet dynamics in the early postoperative phase remains limited (6). Whether dynamic platelet trajectories improve risk stratification beyond static postoperative counts in ATAAD remains unknown.

This retrospective cohort study included 773 patients undergoing surgery for ATAAD. Growth mixture modeling (GMM) was used to identify distinct platelet count trajectories during the first 7 postoperative days. We aimed to explore the independent association between each trajectory phenotype and all-cause mortality and develop a model for early prediction of trajectory phenotype, thereby providing evidence for personalized postoperative management.

## Methods

### Study population

We retrospectively reviewed the medical records of 1,049 consecutive adult patients who underwent surgical repair for ATAAD at the Department of Cardiac Surgery, Nanjing Drum Tower Hospital, between January 2011 and December 2019. The exclusion criteria were: 1) Patients who died intraoperatively or within the first 7 postoperative days, 2) Patients with no platelet tests during ICU stay, 3) Patients with missing preoperative platelet count data and 4) Patients with hematological diseases, liver cirrhosis, or diseases such as chronic thrombocytopenia. Ultimately, 773 patients were included in this retrospective study. The detailed procedure of inclusion and exclusion criteria is visually presented in a flowchart Figure 1.

**Figure 1:**
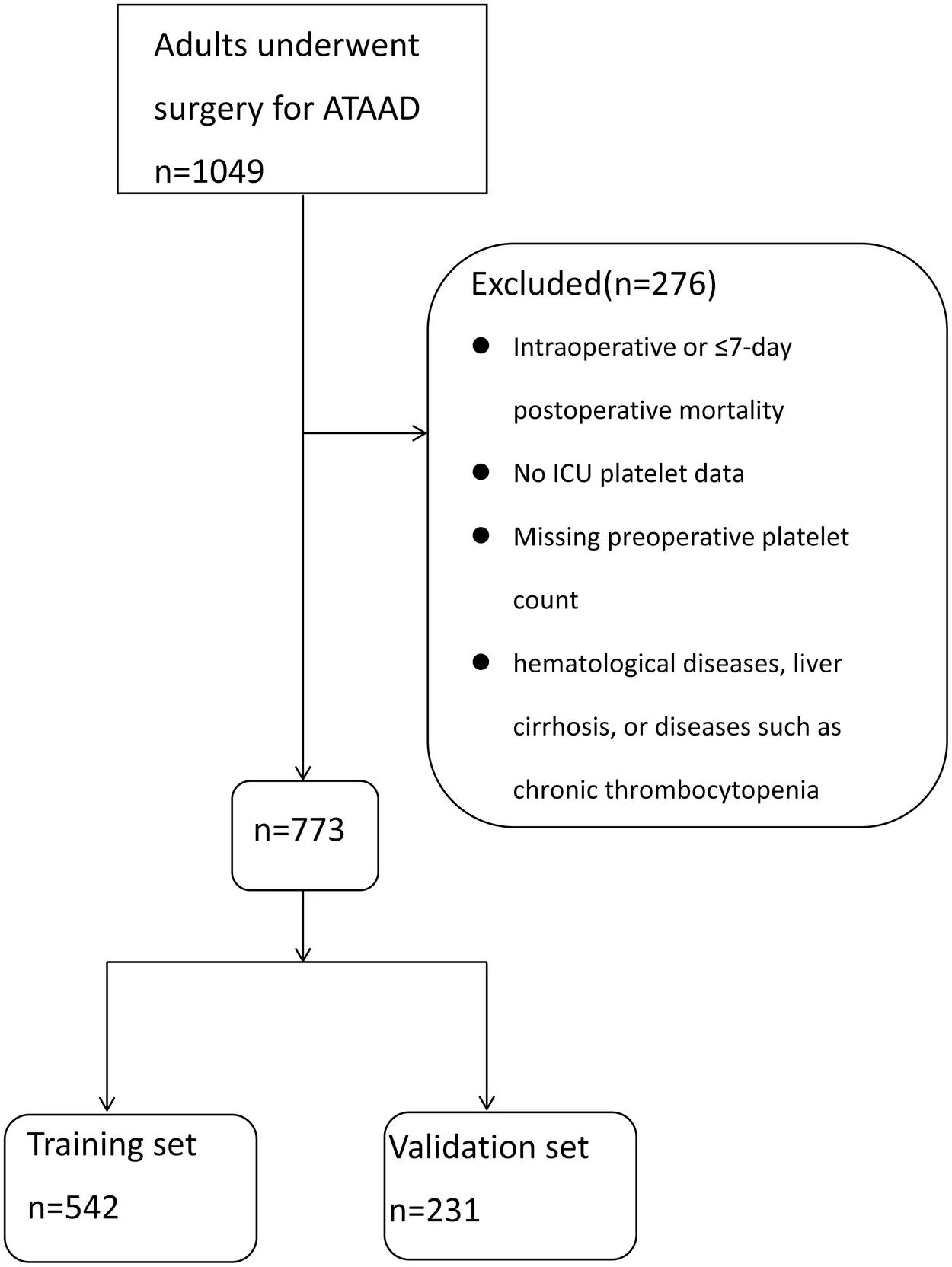
Flowchart of patient selection.

### Variables

Baseline data were collected, including demographic characteristics, medical history, laboratory test results, and intraoperative variables. Platelet counts obtained from postoperative day 1 to day 7 were extracted for trajectory analysis. Patients who died within the first 7 postoperative days were excluded from the trajectory analysis because their platelet series were truncated by death and a 7-day trajectory could not be defined. We recognize that this exclusion may induce survivorship bias, which will be addressed in the Limitation section.

### Outcomes

The primary endpoint was short-term all-cause mortality, defined as death from any cause between postoperative days 7 and 30. The secondary endpoint was long-term all-cause mortality, defined as death from any cause, whether in hospital or after discharge, from surgery through the end of follow-up on 1 August 2024.

### Statistical analysis

The raw data were systematically cleaned to identify and manage outliers. Variables with less than 20% missing data were imputed using the random forest algorithm; variables with 20% or more missing data were excluded. Platelet counts and outcome variables were not imputed. GMM was used to identify distinct longitudinal platelet count trajectories (15, 16). In this study, GMM was constructed using serial platelet count data within the first seven postoperative days to determine the optimal number and morphological patterns of platelet trajectories (17). The optimal number of classes was identified using the following statistical criteria: Bayesian Information Criterion, Akaike Information Criterion, entropy (≥0.80), all classes contained ≥5% of the study sample, the average posterior probability of class membership ≥0.80, and the Lo–Mendell–Rubin likelihood ratio test (LMR-LRT). We also considered interpretability using subject-matter knowledge (18). Based on their patterns of change, each trajectory phenotype was assigned a name. Baseline clinical variables were compared across distinct trajectory groups. Continuous variables were expressed as mean ± standard deviation or median (interquartile range) and were compared among the trajectory groups using one-way analysis of variance or the Kruskal–Wallis test, respectively. Categorical variables were summarized as frequencies and percentages and were compared using the *χ*^2^ test or Fisher's exact test, as appropriate. All statistical tests were two-sided, and *P* < 0.05 was considered statistically significant. All statistical analyses were performed using R software version 4.6.0 (R Foundation for Statistical Computing, Vienna, Austria).

The survival outcome was postoperative 7–30-day all-cause mortality. Kaplan–Meier survival curves were constructed for each of the previously assigned trajectory groups. In addition, Kaplan–Meier survival curves for long-term all-cause mortality were constructed for the three trajectory groups, and between-group differences were compared using the log-rank test. Finally, we determined platelet count phenotypes by comparing differences in 7–30-day survival outcomes derived from K–M curves. They were utilized as the predictive outcome, with general demographic and clinical characteristics serving as predictors, to construct a prediction model which can identification of patients with high-risk platelet count trajectories. The study cohort was randomly divided into training (*n* = 542) and validation (*n* = 231) cohorts at a 7:3 ratio. With trajectory phenotype as the outcome, all candidate baseline variables were entered into a least absolute shrinkage and selection operator (LASSO) regression model in the training cohort (19). Variables retained with a selection frequency ≥70% were ultimately used to construct the logistic regression model. Model performance was comprehensively evaluated in both the training and validation cohorts.

### Ethic

The study protocol was approved by the Ethics Committee of Nanjing Drum Tower Hospital (IRB 2022-157-01). Given the retrospective nature of the study, the requirement for individual consent was waived. The collection of clinical information from the patient population adhered strictly to the guidelines set forth in the Declaration of Helsinki (seventh revision, 2013). The work adheres to the strengthening the reporting of cohort, cross-sectional and case-control studies in surgery (STROCSS) criteria (20).

## Results

### Three-cluster trajectory model

GMM analysis revealed that three-cluster trajectory model provided the best fit, which effectively represented distinct longitudinal patterns of platelet count in ATAAD patients (Figure 2). The three-class nonlinear model achieved an entropy of 0.84, with average posterior probabilities ≥0.90 for all classes. The sample proportions for the three trajectory classes were 50.45%, 35.19%, and 14.36%, respectively, each exceeding the 5% threshold (21, 22), thus indicating satisfactory classification quality. The LMR-LRT indicated that the three-class model was significantly superior to the two-class model (*P* *<* 0.001) (18). Although the four-class model yielded lower information criteria, it contained classes comprising less than 5% of the sample and was therefore not retained(Table 1). Based on a comprehensive evaluation of these criteria, the three-class nonlinear trajectory model with quadratic terms was selected as the optimal model to characterize the dynamic trajectories of platelet counts within the first 7 postoperative days.

**Figure 2:**
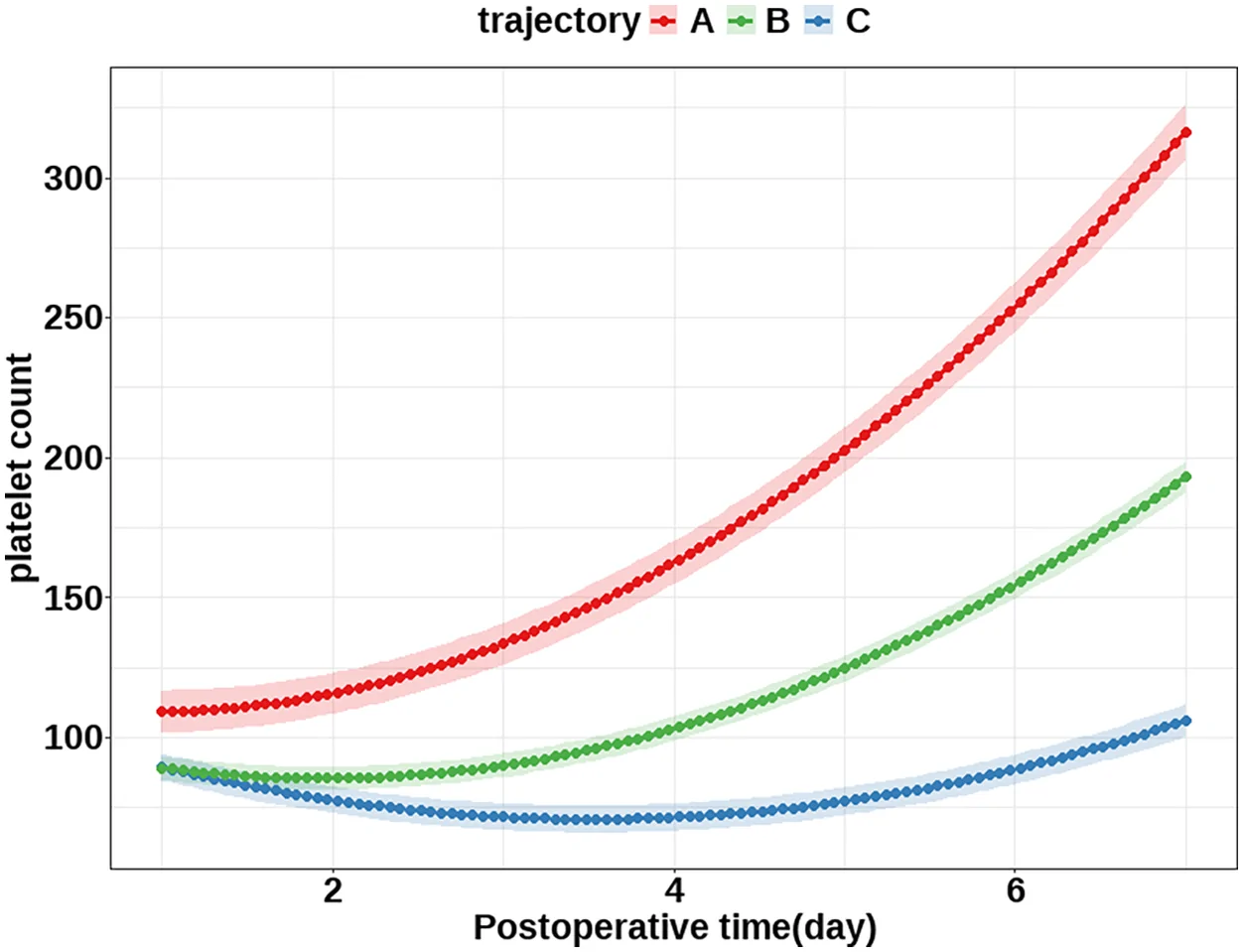
Platelet Count Trends within 7 Postoperative days Across Three Trajectory (*n* = 773). The shaded bands indicate the 95% confidence intervals of the trajectory estimates.

**Table 1:** Effectiveness of trajectory model fitting for platelet count within 7 days after surgery.

ng	AIC	BIC	Entropy	Relative entropy	LMR-LRT test P	Class percentages (%)					Average posterior probability				
						1	2	3	4	5	1	2	3	4	5
Linear															
2	48765.80	48798.35	0.81	0.83	**<0**.**001**	30.79	69.21	0.93	0.95						
3	48353.62	48400.12	0.78	0.75	**<0**.**001**	12.94	38.03	49.03	0.93	0.90	0.86				
4	48285.42	48345.87	0.74	0.59	**<0**.**001**	30.79	6.34	17.72	45.15	0.88	0.88	0.79	0.83		
5	48203.16	48277.57	0.79	0.62	**<0**.**001**	1.55	5.31	31.44	44.24	17.46	0.895	0.90	0.89	0.85	0.80
Quadratic															
2	47273.31	47315.16	0.84	0.86	**<0**.**001**	62.61	37.39	0.96	0.94						
3	46512.67	46573.13	0.84	0.82	**<0**.**001**	50.45	14.36	35.19	0.91	0.94	0.94				
4	46254.56	46333.62	0.81	0.71	**<0**.**001**	24.19	40.24	7.63	27.94	0.86	0.86	0.93	0.93		
5	46133.63	46231.28	0.79	0.65	**<0**.**001**	6.60	37.39	6.99	20.82	28.20	0.89	0.83	0.95	0.87	0.85

ng: number of trajectory group sets; AIC, BIC, Akaike Information Criterion, Bayesian Information Criterion, lower values indicate better model fit; Entropy: a measure of classification quality based on individual posterior probabilities; values closer to 1 indicate clearer classification and higher quality. Relative entropy: the larger means the better the quality of trajectory classification; LMR-LRT test: Lo-Mendell-Rubin Likelihood Ratio Test, used to compare the goodness of fit between k-group and (k + 1)-group models; *P* < 0.05 indicates that the (k + 1)-group model fits significantly better than the k-group model(k:2–5).

The three trajectories identified from the optimal model were designated as TrajeA, TrajeB, and TrajeC. As illustrated in Figure 2, TrajeA (14.36%) exhibited a slow increase in platelet counts during the early postoperative period (days 1–3), followed by an accelerated upward trend during the middle-to-late postoperative period (days 4–7); TrajeB (50.45%) demonstrated a sustained, gradual upward trend throughout the entire observation period without notable acceleration or plateau; and TrajeC (35.19%) showed a mild initial decline, followed by a persistently low and stable level without significant recovery. Overall, 773 patients were included in this retrospective study. Baseline demographic, clinical, and procedural characteristics according to trajectory group are presented in Table 2. Patients in TrajeC (*n* = 272) were significantly older than those in TrajeA (*n* = 111) (57.12 ± 13.46 vs. 45.71 ± 10.82 years, *P* < 0.001), with TrajeB (*n* = 390) falling in between, and the proportion of male patients decreased progressively from TrajeA to C (90.09% vs. 75.64% vs. 69.12%, *P* < 0.001). Compared with TrajeA, TrajeC had a higher burden of preoperative comorbidities and malperfusion, including end-stage renal disease (6.25% vs. 0%, *P* < 0.001), preoperative cerebral ischemia (11.40% vs. 1.80%, *P* = 0.011), coronary artery involvement (23.53% vs. 17.12%, *P* = 0.027), preoperative hypotension (9.19% vs. 2.70%, *P* = 0.049), and cardiac tamponade (13.60% vs. 5.41%, *P* = 0.026). Preoperative hemoglobin (median, 126.00 vs. 138.00 g/L, *P* < 0.001) and platelet count (median, 142.50 vs. 194.00 × 10⁹/L, *P* < 0.001) were lowest in Traje C, whereas prothrombin time (PT), international normalized ratio (INR), aspartate aminotransferase (AST), creatinine, and blood urea nitrogen increased stepwise from TrajeA to TrajeC (all *P* < 0.05). Intraoperatively, TrajeC underwent longer operations (8.01 ± 1.89 vs. 7.33 ± 1.67 h, *P* = 0.002), with longer cardiopulmonary bypass (233.50 vs. 206.00 min, *P* = 0.002) and aortic cross-clamp times (169.92 vs. 156.01 min, *P* = 0.042), and received significantly greater volumes of red blood cells, plasma, cryoprecipitate, platelets, and total transfusions (all *P* < 0.01). Postoperatively, the incidence of stroke (9.56% vs. 2.70%, *P* = 0.006), neurologic dysfunction (10.66% vs. 2.70%, *P* = 0.003), acute renal failure (42.65% vs. 18.02%, *P* < 0.001), continuous renal replacement therapy (25.00% vs. 1.80%, *P* < 0.001), and surgical site infection (4.78% vs. 0%, *P* = 0.029), as well as the proportion of patients with a hospital stay exceeding 30 days (22.06% vs. 11.71%, *P* = 0.003), were highest in Traje C, with Traje B showing intermediate values for nearly all variables.

**Table 2:** Baseline Characteristics of the three platelet trajectory groups.

Variable	Trajectory			Statistic	P value
	Traje A( n = 111)	Traje B( n = 390)	Traje C(n = 272)		
Age, yr	45.71 ± 10.82	51.62 ± 12.22	57.12 ± 13.46	H = 64.94	<0.001
Gender, Male,n (%)	100 (90.09%)	295 (75.64%)	188 (69.12%)	*χ*^2^ = 18.724	<0.001
BMI, kg/m^2^	26.73 ± 4.12	26.08 ± 4.32	25.41 ± 4.17	F = 4.320	0.014
Smoke, *n* (%)	39 (35.14%)	97 (24.87%)	58 (21.32%)	χ^2^ = 8.021	0.018
Alcohol, *n* (%)	30 (27.03%)	67 (17.18%)	41 (15.07%)	χ^2^ = 7.924	0.019
Hypertension, *n* (%)	72 (64.86%)	285 (73.08%)	209 (76.84%)	χ^2^ = 5.772	0.056
Diabetes, *n* (%)	0 (0.00%)	19 (4.87%)	7 (2.57%)	-	0.020
End-stage renal disease, *n* (%)	0 (0.00%)	2 (0.51%)	17 (6.25%)	-	<0.001
Cerebralischemia preoperative, *n* (%)	2 1.80%)	37 (9.49%)	31 (11.40%)	χ^2^ = 8.991	0.011
Limbischemia preoperative, *n* (%)	14 (12.61%)	49 (12.56%)	42 (15.44%)	χ^2^ = 1.234	0.540
Bowelischemia Preoperative, *n* (%)	2 (1.80%)	17 (4.36%)	14 (5.15%)	-	0.337
Cororyartery involvement, *n* (%)	19 (17.12%)	60 (15.38%)	64 (23.53%)	χ^2^ = 7.215	0.027
Preoperative hypotension, *n* (%)	3 (2.70%)	23 (5.90%)	25 (9.19%)	χ^2^ = 6.012	0.049
Cardiac tamponade, *n* (%)	6 (5.41%)	34 (8.72%)	37 (13.60%)	χ^2^ = 7.263	0.026
Preoperative platelet	194.00 (154.00,226.00)	155.00 (126.75,187.00)	142.50 (108.00,175.75)	H = 61.182	<0.001
Preoperative white blood cell	12.80 (10.60,15.75)	11.75 (9.40,14.34)	11.40 (8.88,14.60)	H = 5.955	0.051
Preoperative hemoglobin	138.00 (128.00,149.25)	133.00 (118.00,145.00)	126.00 (113.75,141.00)	H = 27.706	<0.001
Preoperative PT	11.95 (11.15,12.90)	12.10 (11.20,13.34)	12.55 (11.60,14.10)	H = 16.259	<0.001
Preoperative APTT	28.00 (26.33,31.65)	28.15 (26.00,33.90)	29.80 (25.90,36.20)	H = 4.501	0.105
Preoperative INR	1.04 (0.97,1.13)	1.05 (0.98,1.17)	1.09 (1.01,1.22)	H = 15.164	<0.001
Preoperative AST	33.00 (23.00,45.00)	31.00 (21.85,60.25)	38.35 (24.00,89.79)	H = 12.597	0.002
Preoperative Cr	76.00 (64.45,93.85)	78.05 (59.95,107.80)	91.05 (66.81,133.81)	H = 19.559	<0.001
Preoperative BUN	6.30 (5.43,7.70)	6.61 (5.23,8.38)	7.15 (5.63,9.02)	H = 10.857	0.004
Procedural characteristics					
Root surgery, *n* (%)				-	0.508
Root Bentall	19 (17.12%)	76 (19.49%)	58 (21.32%)		
Root reconstruction	87 (78.38%)	306 (78.46%)	209 (76.84%)		
untreated	5 (4.50%)	8 (2.05%)	5 (1.84%)		
Arch surgery, *n* (%)				-	0.722
Hemi arch replacement	22 (19.82%)	68 (17.44%)	44 (16.18%)		
Total arch replacement with FET	51 (45.95%)	193 (49.49%)	122 (44.85%)		
Arch stent	37 (33.33%)	125 (32.05%)	101 (37.13%)		
Untreated	1 (0.90%)	4 (1.03%)	5 (1.84%)		
CABG, *n* (%)	7 (6.31%)	16 (4.10%)	24 (8.82%)	χ^2^ = 6.266	0.044
Operation time(hours)	7.33 ± 1.67	7.69 ± 1.71	8.01 ± 1.89	F = 6.39	0.002
CPB (min)	206.00 (181.50,250.00)	221.50 (187.00,264.00)	233.50 (194.00,277.00)	H = 12.927	0.002
Aortic clamp(min)	156.01 ± 50.65	162.73 ± 49.62	169.92 ± 55.60	F = 3.184	0.042
Intraoperative transfusion volume					
RBCs	4.00 (3.00,6.00)	5.00 (3.50,8.00)	6.00 (4.00,10.00)	H = 26.555	<0.001
Plasma	700.00 (525.00,950.00)	812.50 (600.00,1000.00)	875.00 (618.75,1006.25)	H = 13.255	0.001
Cryo	10.00 (7.00,13.00)	10.00 (8.00,13.00)	12.00 (9.25,15.25)	H = 23.090	<0.001
PC	1.00 (1.00,2.00)	1.00 (1.00, 2.00)	2.00 (1.00,2.00)	H = 32.953	<0.001
Total	2225.00 (1478.12,2875.00)	2550.00 (1950.00,3298.44)	2968.75 (2168.75,3935.94)	H = 35.638	<0.001
Postoperative data					
Intracranial hemorrhage, *n* (%)	0 (0.00%)	4 (1.03%)	4 (1.47%)	-	0.533
Stroke, *n* (%)	3 (2.70%)	17 (4.36%)	26 (9.56%)	χ^2^ = 10.185	0.006
Neurologic Dysfunction, *n* (%)	3 (2.70%)	19 (4.87%)	29 (10.66%)	χ^2^ = 11.908	0.003
Paraplegia, *n* (%)	1 (0.90%)	6 (1.54%)	8 (2.94%)	-	0.400
Gl bleeding, *n* (%)	0 (0.00%)	5 (1.28%)	5 (1.84%)	-	0.478
Bowel ischemia, *n* (%)	0 (0.00%)	5 (1.28%)	5 (1.84%)	-	0.459
Limb ischemia, *n* (%)	1 (0.90%)	6 (1.54%)	8 (2.94%)	-	0.427
Surgical site infection, *n* (%)	0 (0.00%)	11 (2.82%)	13 (4.78%)	-	0.029
ARF, *n* (%)	20 (18.02%)	145 (37.18%)	116 (42.65%)	χ^2^ = 20.900	<0.001
CRRT, *n* (%)	2 (1.80%)	26 (6.67%)	68 (25.00%)	χ^2^ = 62.950	<0.001
Re-exploration, *n* (%)	3 (2.70%)	24 (6.15%)	25 (9.19%)	χ^2^ = 5.701	0.058
Length of hospital stay >30 days, *n* (%)	13 (11.71%)	50 (12.82%)	60 (22.06%)	χ^2^ = 11.930	0.003

1. F: ANOVA; χ^2^: Chi-square test; -: Fisher's exact test; H: Kruskal–Wallis H test.

2. Categorical variables were presented as number (percentage). For continuous variables, the mean, standard deviation and median were rounded to two decimal places, and the *P* value was rounded to three decimal places. Normally distributed continuous variables were expressed as Mean ± SD, while skewed variables were presented as Median (IQR). ANOVA was applied to data satisfying normality and homoscedasticity, while Kruskal–Wallis test was adopted otherwise.

3. BMI, body mass index; PT, prothrombin time; APTT, activated partial thromboplastin time; INR, international normalized ratio; AST, aspartate aminotransferase; Cr, serum creatinine; BUN, blood urea nitrogen; Total arch replacement with FET, total arch replacement with frozen elephant trunk; CABG coronary artery bypass grafting; CPB, cardiopulmonary bypass; RBCs, red blood cell transfusion; Plasma, plasma transfusion; Cryo, cryoprecipitate; PC, platelet concentrate; Total, total transfusion volume; Gl bleeding, Gastrointestinal bleeding; ARF, acute renal failure; CRRT, continuous renal replacement therapy.

### Outcomes in three latent clusters

We first evaluated the association between platelet trajectory groups and the risk of postoperative 7–30-day mortality. With TrajeB as the reference, TrajeC was associated with a higher mortality risk at follow-up (HR: 5.97, 95% CI: 2.55–13.96, *P* < 0.001) (Figure 3). For TrajeA, no death events were observed, resulting in complete data separation. Pairwise comparisons of postoperative 7–30-day mortality risk were further conducted using the log-rank test and the Max Combo test (Table 3). Survival did not differ significantly between TrajeA and TrajeB (log-rank: *χ^2^* = 2.009, *P* = 0.160; Max Combo: *Z* = 1.417, *P* = 0.233). In contrast, the survival curves between Traje C and Traje B differed significantly (log-rank: *χ^2^* = 33.726, *P* < 0.001; Max Combo: *Z* = 5.829, *P* < 0.001). Synthesizing the postoperative 7–30-day mortality risks and Cox regression results across the three trajectory groups, the platelet count trajectories were consolidated into phenotypic classifications: TrajeA (no observed deaths) and TrajeB (few deaths) were merged into the low-risk phenotype, whereas TrajeC was designated as the high-risk platelet count phenotype. The merging was based on the following considerations. First, the primary outcome of this study was postoperative 7–30-day mortality, within which TrajeA and TrajeB had similarly low event rates, suggesting comparable short-term risk between the two groups. Second, as the model was intended for the rapid identification of patients at high risk of short-term mortality in the early perioperative period, the merged binary outcome categories offered greater simplicity, thereby improving model stability and clinical applicability. In the subsequent Cox regression analysis based on the binary phenotype, patients with the high-risk phenotype had a significantly higher risk of postoperative 7–30-day mortality than those with the low-risk phenotype (HR: 7.28, 95% CI: 3.09–17.20, *P* < 0.001; Table 4). In addition, Kaplan–Meier analysis revealed significant differences in long-term survival among the three trajectory groups (overall log-rank *P* < 0.05; Table 5 and Figure 4).

**Figure 3:**
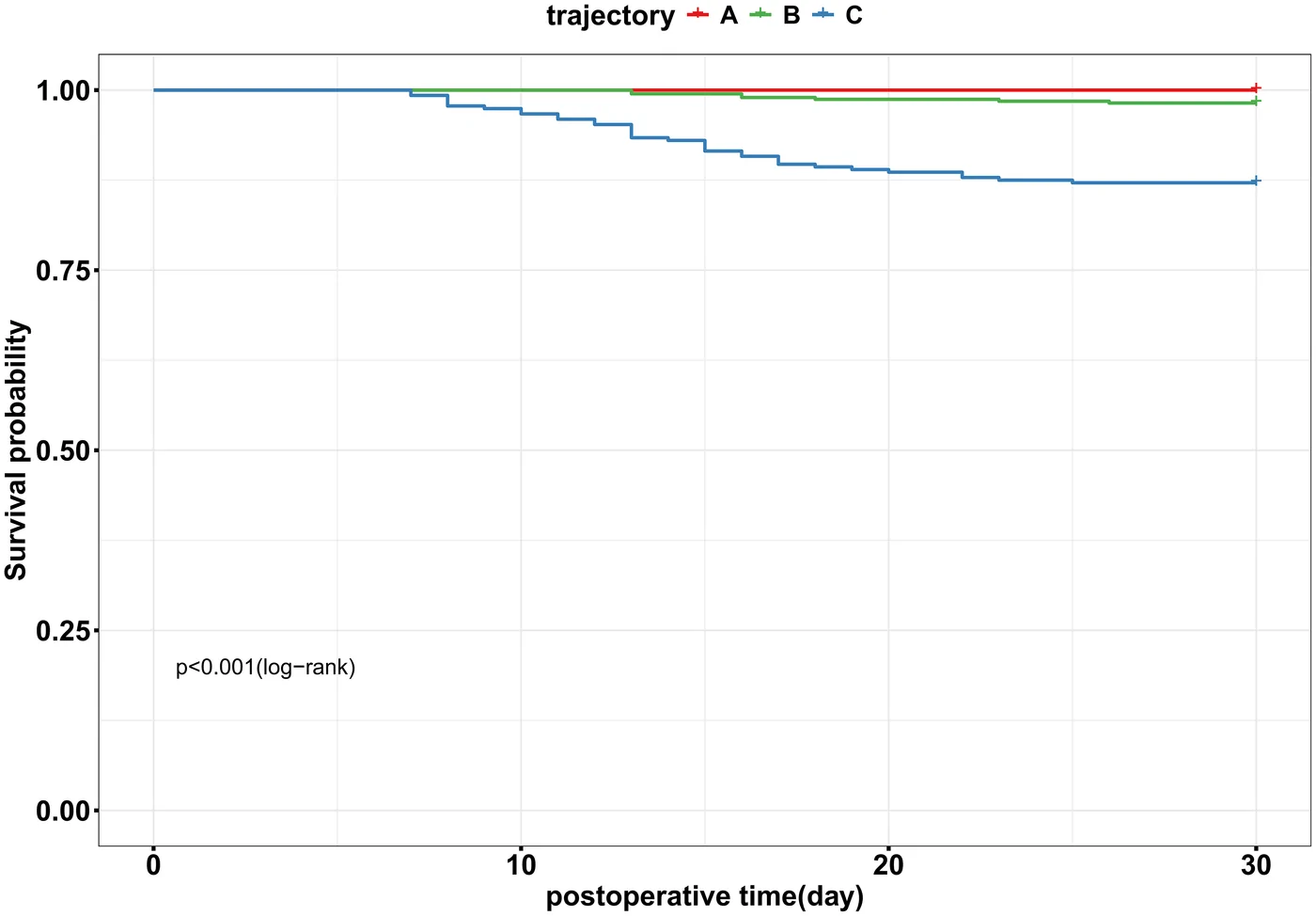
Kaplan–Meier Survival Curves During Postoperative Days 7–30. Survival curves were plotted using the Kaplan–Meier method. The horizontal axis represents postoperative time (in days), and the vertical axis represents survival probability. The log-rank test was used to compare differences between the two survival curves.

**Table 3:** Results of pairwise log-rank and max combo comparisons among different trajectory groups 7-30-day postoperative survival curve.

Trajectory	Log rank	Max Combo		
	Statistic	Log rank *P*	Statistic	Max Combo *P*
B				
A	χ^2^ = 2.009	0.160	Z = 1.417	0.233
C	χ^2^ = 33.726	**<0.001**	Z = 5.829	**<0**.**001**

1. TrajeB is the reference group.

2. Pairwise comparisons among trajectory groups were performed using Log-rank test and Max Combo test. A two-sided *P* value < 0.05 was considered statistically significant.

3. Bold values indicate statistical significance (*P* < 0.001).

**Table 4:** Cox Regression analysis of postoperative mortality between days 7 and 30.

Subgroup	Sample size	Survival number	Number of deaths	median follow-up time (day)	HR(95%CI)	P
Trajectory						
B	390	383 (98.21%)	7 (1.79%)	30		
A	111	111 (100.00%)	0 (0.00%)	30	-(-, -)	0.995
C	272	237 (87.13%)	35 (12.87%)	30	5.97 (2.55-13.96)	**<0**.**001**
Phenotype						
Low Risk	501	494 (98.60%)	7 (1.40%)	30		
High Risk	272	237 (87.13%)	35 (12.87%)	30	7.28 (3.09-17.20)	**<0**.**001**

1. Traje B was the reference for Cox analysis of trajectory groups, and the low-risk phenotype was the reference for Cox analysis of different phenotypes.

2. Number of deaths: deaths occurring between postoperative days 7 and 30.

3. HR: Hazard ratio. An HR > 1 indicates a higher risk of death compared with the reference group; A *P*-value < 0.05 was considered statistically significant; -: The incidence of TrajeA is 0, leading to an abnormal HR value.

4. Both models included the following variables for covariate adjustment: Age, Gender, BMI, End-stage renal disease, Smoke, Preoperative hypotension, Malperfusion syndrome, Tampode, CABG, Operation time hours, CPBmin, and Total.

5. Bold values indicate statistical significance (*P* < 0.001).

**Table 5:** Results of pairwise log-rank comparisons among different trajectory groups long-term postoperative survival curve.

Trajectory	Log rank	
	Statistic	Log rank P
B		
A	χ^2^ = 4.82	0.028
C	χ^2^ = 48.41	**<0**.**001**

1. TrajeB is the reference group.

2. Pairwise comparisons among trajectory groups were performed using Log-rank test. A two-sided *P* value < 0.05 was considered statistically significant.

**Figure 4:**
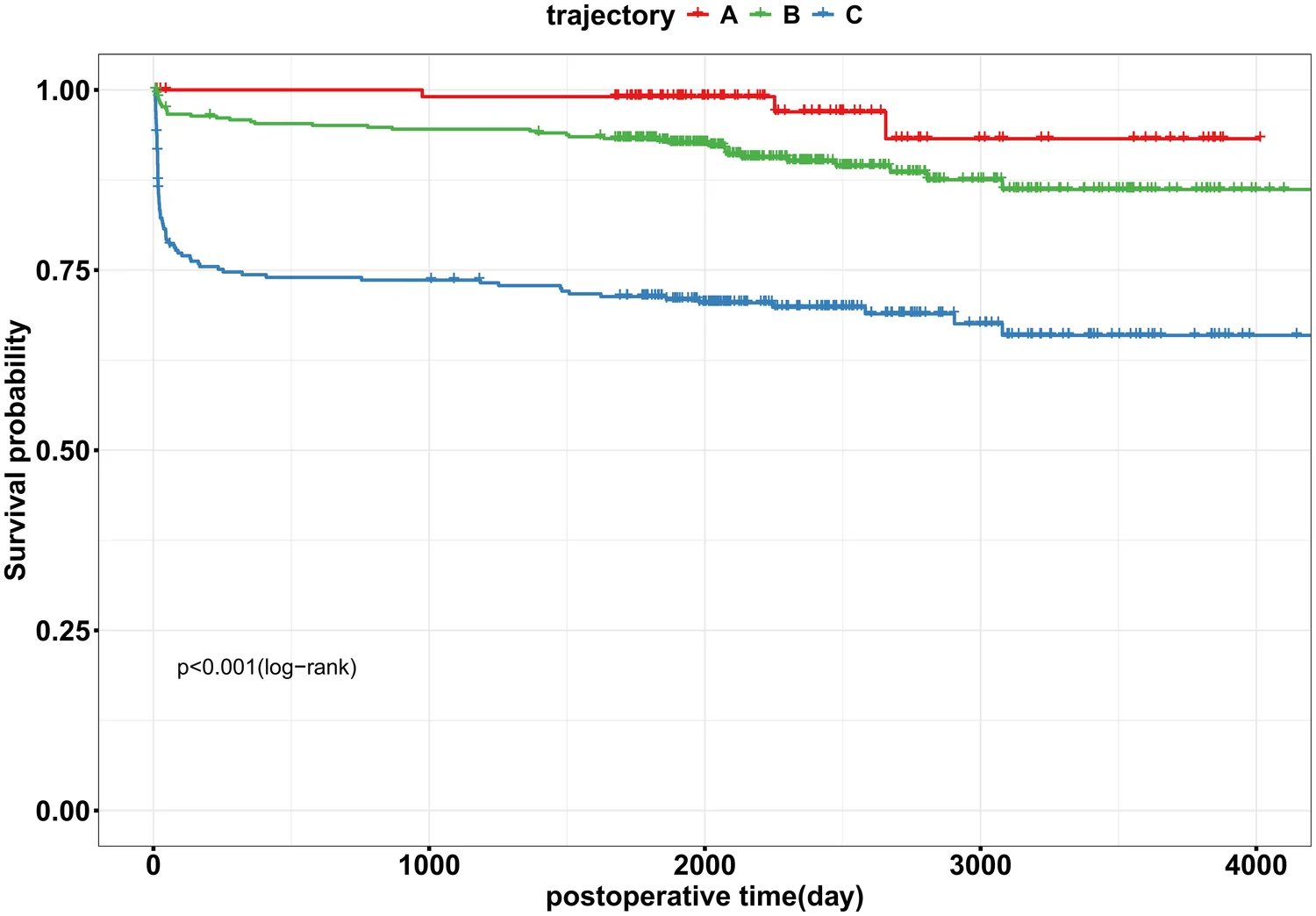
Kaplan–Meier Survival Curves for Long-term Postoperative Survival. Survival curves were plotted using the Kaplan–Meier method. The horizontal axis represents postoperative time (in days), and the vertical axis represents survival probability. The log-rank test was used to compare differences between the two survival curves.

### Construct a prediction model

The training cohort comprised 542 patients, of whom 191 (35.24%) had the high-risk phenotype, and the validation cohort comprised 231 patients, of whom 81 (35.06%) had the high-risk phenotype. As shown in supplement Table 1, no significant differences were observed for any variables between the training and validation cohort (*P* > 0.05), indicating satisfactory baseline comparability following randomization. Besides, supplement Table 2 presents the collinearity assessment results following variable exclusion, with no severe multicollinearity detected among the 27 candidate variables. Bootstrap LASSO regression was applied to the 27 candidate variables: in each of the 1,000 bootstrap resamples, a LASSO model with 10-fold cross-validation was fitted and variables with non-zero coefficients at lambda.1se were recorded; variables selected in ≥70% of the resamples were retained as final predictors. This procedure yielded five predictors: age, preoperative creatinine, total transfusion volume, end-stage renal disease, and operative duration (Supplementary Table S3). In the training cohort, the model achieved an area under the receiver operating characteristic curve (AUC) of 0.73 (95% CI: 0.69–0.78), with a sensitivity of 0.70, a specificity of 0.59, an accuracy of 0.63, and an F1-score of 0.57. In the validation cohort, the AUC was 0.69 (95% CI: 0.61–0.76), with a sensitivity of 0.65, a specificity of 0.59, an accuracy of 0.61, and an F1-score of 0.54. These results indicate moderate discriminative ability of the model, with comparable performance between the training and validation sets and no evident overfitting (Table 6). Figure 5 illustrates the receiver operating characteristic (ROC) curves for both datasets. Figure 6 presents the nomogram developed from the final logistic regression model for individualized risk prediction of high-risk platelet count phenotypes. Calibration curves demonstrated good agreement between predicted and observed probabilities in the training cohort, with satisfactory calibration maintained in the validation cohort (Figure 7A,B). In the training cohort, the calibration curve showed close approximation to the ideal diagonal line (Brier score = 0.19; Figure 7A). The Hosmer–Lemeshow test was significant in the training cohort (*χ*^2^ = 19.53, *P* = 0.012), which likely reflects its sensitivity to minor deviations in larger samples. In the validation cohort, the calibration curve demonstrated good agreement between predicted probabilities and observed outcomes (Brier score = 0.20; Figure 7B); the Hosmer–Lemeshow test yielded *χ*^2^ = 12.31 (*P* = 0.138), indicating that the model's predicted risks closely matched the actual event probabilities. Although the calibration curve exhibited minor fluctuations in the low-to-moderate probability range, overall calibration remained adequate without significant miscalibration. Decision curve analysis was performed to evaluate the potential clinical utility of the nomogram (Figure 6). In the training set, the prediction model provided a higher net benefit than both the treat-all and treat-none strategies across a wide range of threshold probabilities (approximately 0.25–0.90; Figure 8A), suggesting potential value for model-guided clinical decision-making across most clinically relevant thresholds. Consistent findings were observed in the validation set, where the model maintained a clear net benefit over the two default strategies within a comparable threshold range (approximately 0.10–0.75), although the net benefit approached zero at very high thresholds (>0.80) (Figure 8B). These results suggest that the nomogram has favorable clinical applicability for identifying patients with high-risk platelet trajectories.

**Table 6:** Evaluation metrics for logistic regression prediction model.

Data Set	Sensitivity	Specificity	AUC(95%CI)	F1-Score	Accuracy
Train	0.70	0.59	0.73 (0.69, 0.78)	0.57	0.63
Test	0.65	0.59	0.69 (0.61, 0.76)	0.54	0.61

This table summarizes the performance metrics of the logistic regression prediction model, which predicts the platelet count phenotype as the outcome. The metrics include sensitivity, specificity, the Area under the Curve (AUC) with a 95% confidence interval, F1-Score, and accuracy for both the training and test datasets. These metrics assess the model's ability to differentiate between different platelet count phenotypes (e.g., high-risk and low-risk), with the AUC representing the model's discriminative power. A higher AUC indicates better performance in classifying the platelet count phenotypes.

**Figure 5:**
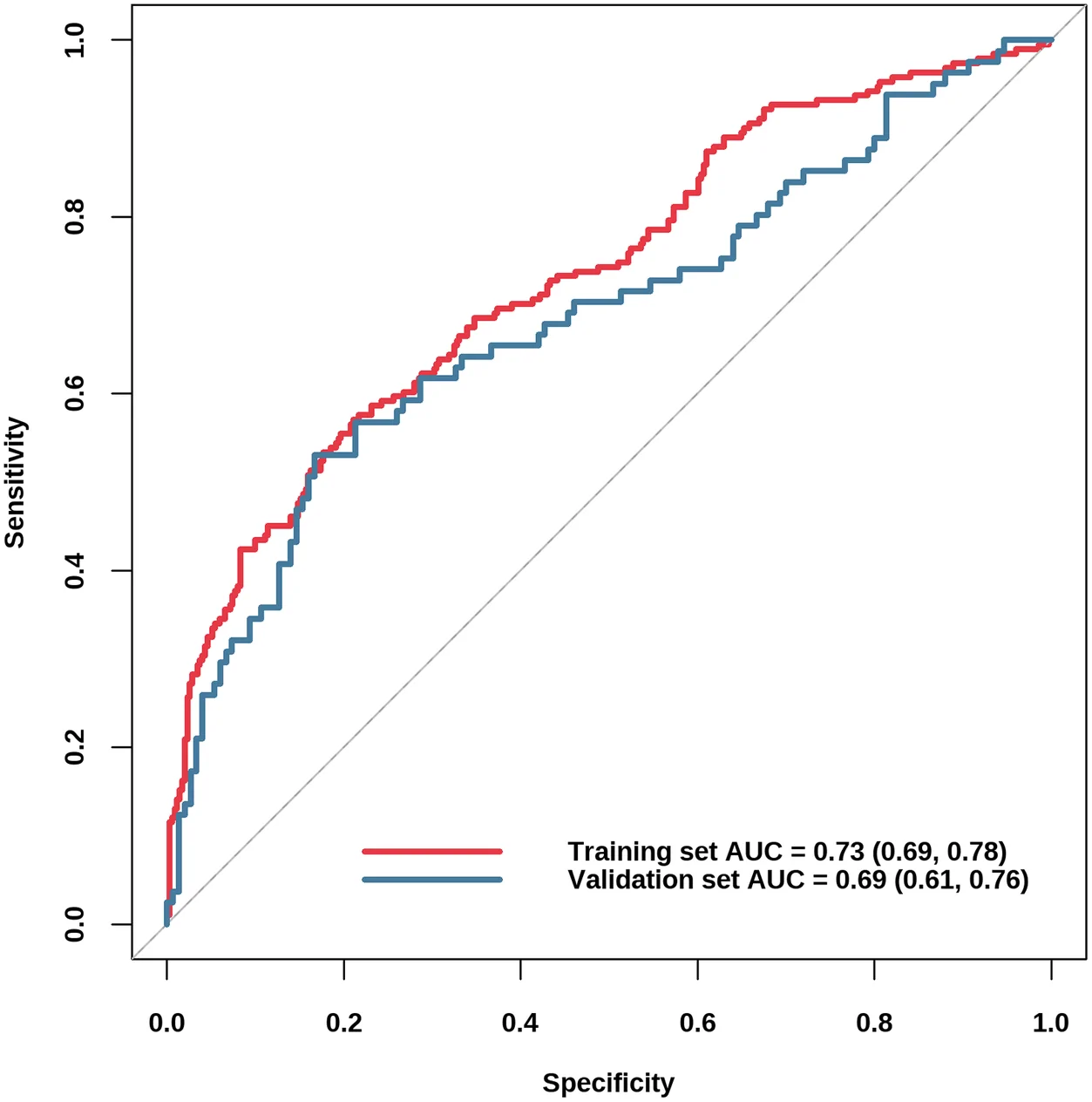
ROC curve plot This figure presents the Receiver Operating Characteristic (ROC) curves for both the training cohort and the validation cohort. The ROC curve helps evaluate the predictive performance of the logistic regression model. The area under the curve (AUC) is reported for both datasets, with a higher AUC indicating better model performance.

**Figure 6:**
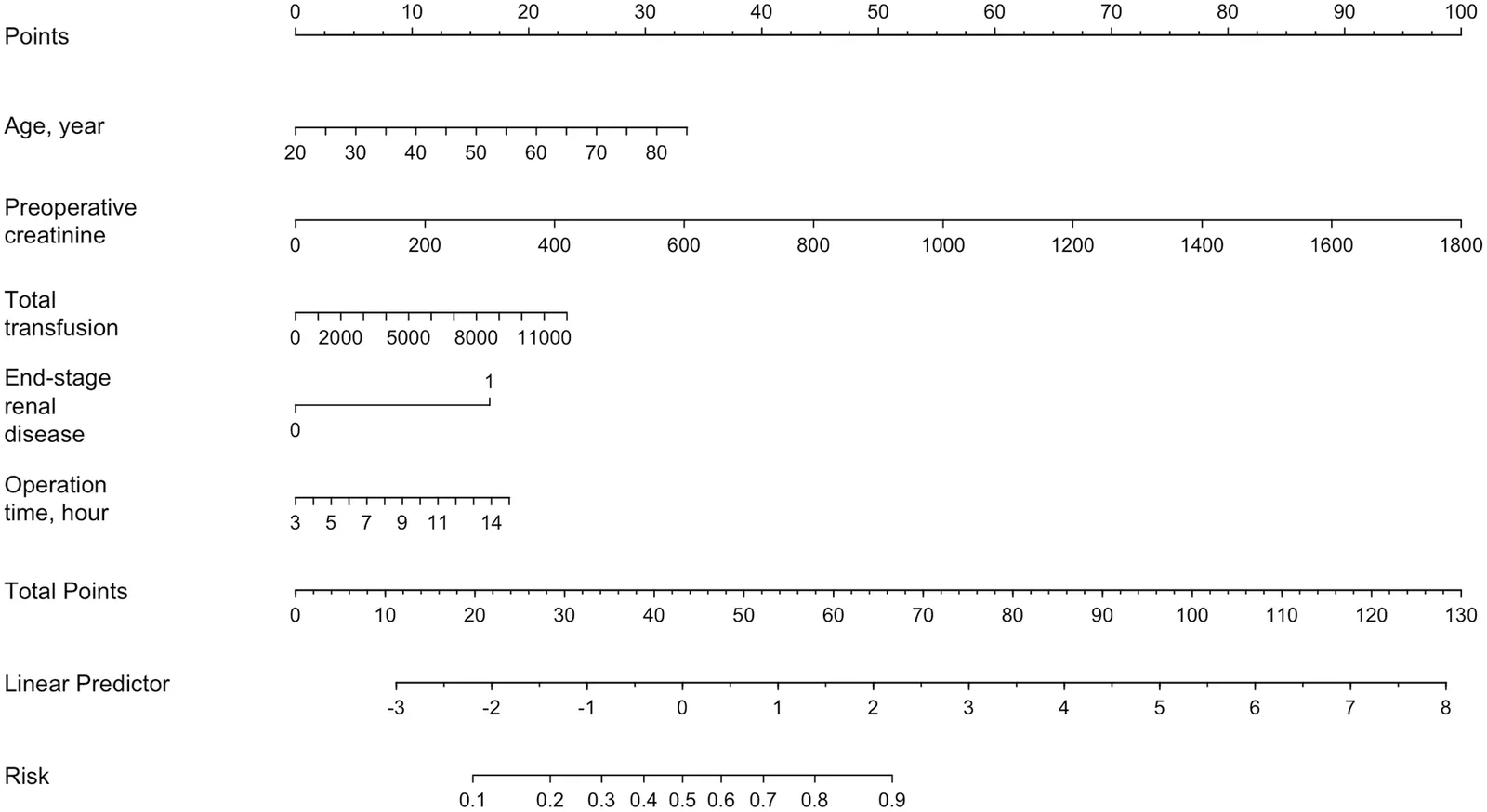
Risk prediction nomogram based on logistic regression prediction model nomogram for predicting the probability of a high-risk postoperative platelet trajectory phenotype.

**Figure 7:**
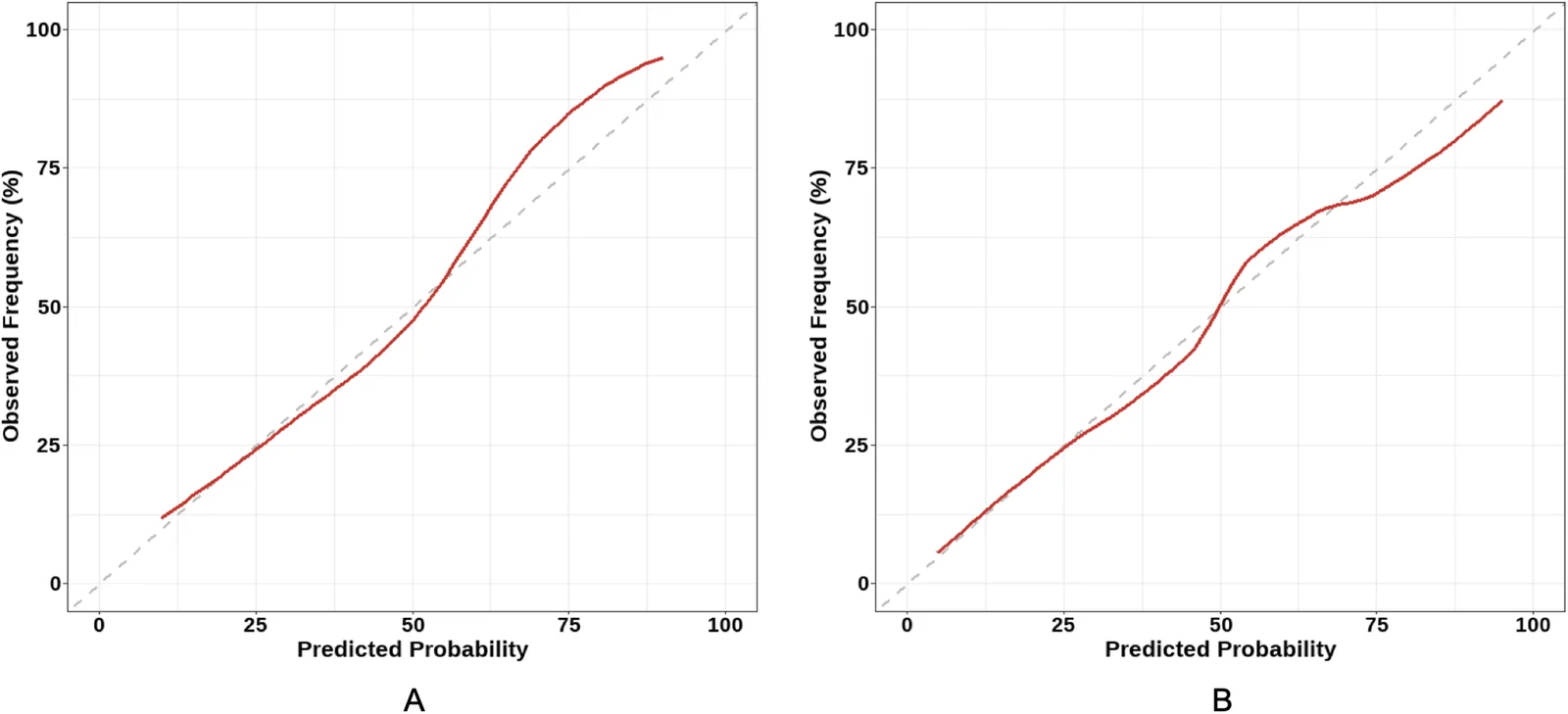
Calibration curves of the training cohort and validation cohort. This figures show the calibration curves for both the training and validation cohorts **(A)** training cohort; **(B)** validation cohort. The x-axis represents the predicted probability of the model, while the *y*-axis represents the observed probability. The closer the curve aligns with the diagonal line, the better the model is calibrated.

**Figure 8:**
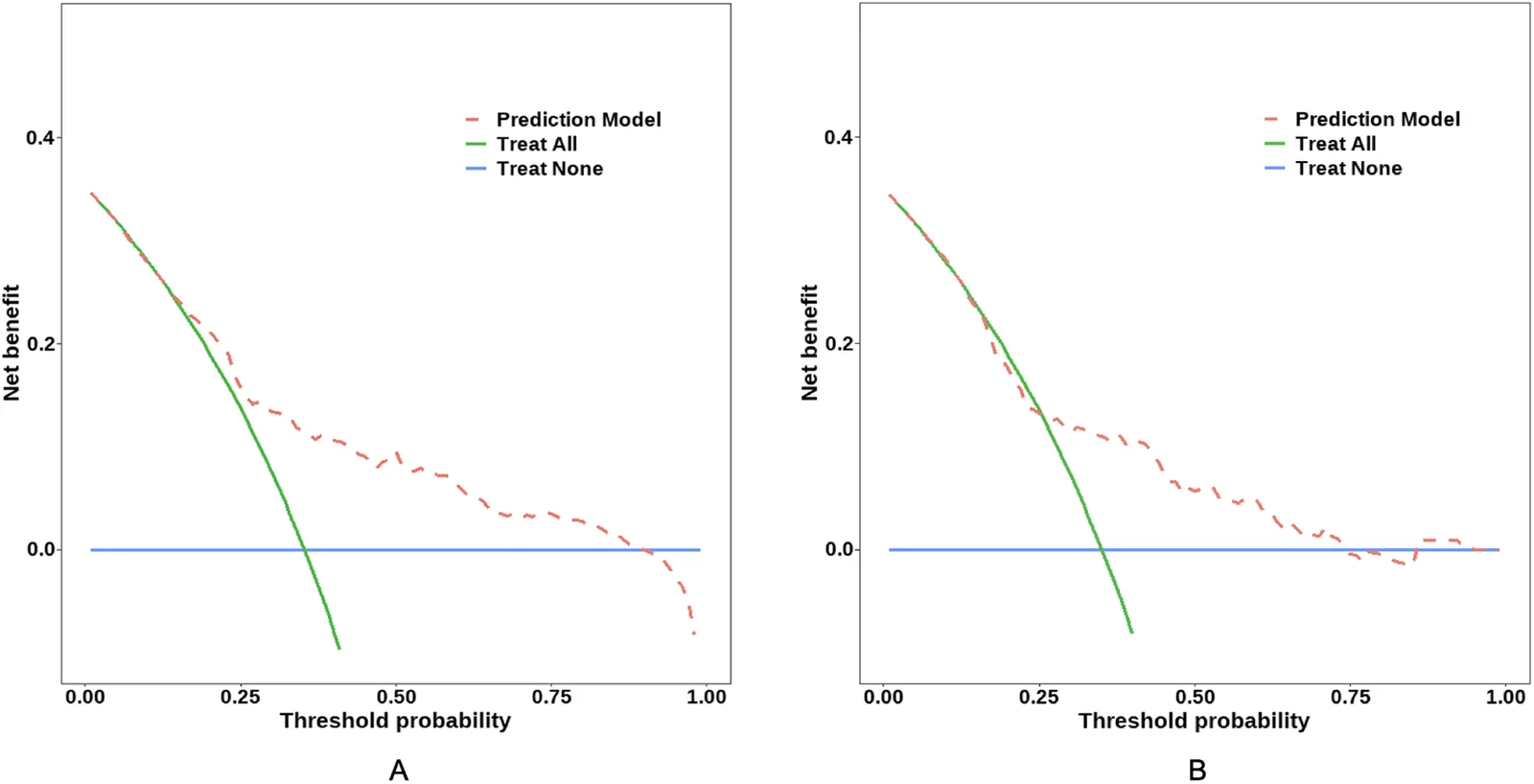
Decision curve analysis (DCA) for the logistic regression model of the training cohort and validation cohort. This figures display the Decision Curve Analysis (DCA) for the logistic regression model **(A)** training cohort; **(B)** validation cohort). The *x*-axis represents the threshold probability, and the *y*-axis represents the net benefit of using the model. The curves show the clinical utility of the model at different threshold values.

Finally, total transfusion volume emerged as an important predictor of the high-risk phenotype (Traje C). However, this measure includes platelet concentrate (PC) transfusions, which can directly alter the measured platelet count. In clinical practice, platelets are typically transfused in response to reduced platelet counts; therefore, this variable may, to some extent, serve as a proxy for the outcome rather than a fully independent predictor, raising the possibility of confounding. To evaluate the impact of this potential confounding on the model, we reconstructed the nomogram after excluding PC from the total transfusion volume. The discrimination and AUC of the model remained largely unchanged (AUC = 0.73; Supplementary Figures 1–S4), suggesting that the predictive performance of the model does not depend on platelet transfusion. Instead, total transfusion volume may serve as a composite marker of the severity of intraoperative bleeding and coagulopathy, with platelet transfusion representing only one component.

## Discussion

This investigation elucidates several pivotal aspects. First, although postoperative thrombocytopenia is prevalent among ATAAD patients after surgical repair, individuals exhibit highly heterogeneous dynamic trends in platelet count recovery. Second, the core focus of this study was to examine the correlation between longitudinal platelet dynamic patterns and both short-term and long-term postoperative mortality in ATAAD patients. Third, taking platelet trajectory phenotype as the predictive outcome and routine demographic and perioperative clinical characteristics as predictive factors, we developed a practical predictive model for high-risk platelet trajectory screening.

In this retrospective cohort of 773 patients undergoing surgical repair of ATAAD, we identified three distinct nonlinear postoperative platelet-count trajectories using GMM and found that these trajectories were significantly associated with both short- and long-term mortality. On this basis, we constructed a logistic regression prediction model incorporating five readily available clinical variables: age, preoperative creatinine, total transfusion volume, end-stage renal disease, and operation time. These findings may facilitate the early identification of high-risk platelet trajectory phenotypes and the formulation of individualized intervention strategies for ATAAD patients.

Postoperative thrombocytopenia is a major clinical challenge in patients undergoing surgical repair of ATAAD. Accumulating evidence has linked postoperative thrombocytopenia to adverse perioperative outcomes following cardiac surgery (5, 12, 13). In a large cohort of 4,217 patients undergoing coronary artery bypass grafting, Kertai et al. (13) showed that a lower postoperative platelet nadir was independently associated with acute kidney injury and short-term all-cause mortality. Specifically focusing on aortic dissection populations, Sandhu et al. (23) reported that sustained platelet decline occurring after postoperative day 4 served as a valuable biomarker for poor 30-day and long-term survival in patients undergoing ATAAD repair. Similarly, Tang et al. (8) enrolled 457 ATAAD patients undergoing median sternotomy; after correcting for selection bias via inverse probability of treatment weighting, they found that patients with a platelet nadir below 75 × 10^3/*μ*Lwithin 72 h postoperatively had a significantly higher long-term mortality rate (37.7% vs. 4.7%). Additionally, platelet levels exhibited a nonlinear negative correlation with mortality risk, with an AUC of 0.769 for predicting mid-term mortality. However, these studies relied solely on static platelet measurements, which inherently fail to capture longitudinal dynamic changes, such as transient recovery followed by secondary decline or delayed platelet recovery, or account for between-patient heterogeneity in recovery. These limitations hinder refined postoperative risk stratification. Accordingly, the present study investigated the prognostic value of longitudinal platelet trajectory patterns following ATAAD surgery.

Using GMM with seven consecutive postoperative platelet measurements, we identified three distinct platelet recovery phenotypes, which may enable more refined prognostic stratification. Patients in the TrajeA and TrajeB groups (64.81%) exhibited a rapid postoperative recovery in platelet counts. These trajectory patterns may suggest a relatively preserved bone marrow hematopoietic reserve and thrombopoietic capacity and may be associated with more favorable outcomes during postoperative days 7–30. In contrast, the TrajeC group (35.19%) maintained persistently low platelet levels without an obvious recovery trend, representing the highest-risk trajectory in this cohort. This persistent thrombocytopenic trajectory was associated with a significantly increased risk of postoperative mortality between days 7 and 30. The persistent thrombocytopenic trajectory observed in Traje C may reflect the cumulative effects of preoperative and intraoperative insults. Preoperatively, patients in TrajeC were older and had significantly higher proportions of end-stage renal disease, cerebral ischemia, coronary artery involvement, hypotension, and cardiac tamponade. They also had the lowest hemoglobin levels and platelet counts, the most prolonged PT, and the highest INR, creatinine, and blood urea nitrogen values. These findings suggest that tissue hypoperfusion, coagulation abnormalities, impaired hepatorenal reserve, and ongoing platelet consumption may already have been present before surgery. Intraoperatively, TrajeC experienced the longest operative, cardiopulmonary bypass, and aortic cross-clamp times, as well as the greatest transfusion volumes of red blood cells, plasma, and platelets. In addition, ischemia–reperfusion injury, CPB-induced inflammation, and hemodilution may have further exacerbated platelet consumption and destruction. The cumulative effects of these preoperative and intraoperative insults may have blunted postoperative platelet recovery in TrajeC, in contrast to the recovery observed in TrajeA and B, resulting in a persistently low, or “stagnant,” trajectory. The “stagnant” platelet pattern indicates sustained platelet consumption or impaired platelet production. Importantly, the higher mortality risk in TrajeC may be related not only to bleeding associated with thrombocytopenia but also to systemic inflammation and progressive organ dysfunction reflected by platelet dynamics.

Platelet trajectories may guide personalized anticoagulation and resource allocation. Patients with high-risk platelet trajectories may require individualized timing of anticoagulation initiation to balance bleeding and thrombotic risks. Additionally, this subgroup should be escalated to a higher level of care early, with consideration of delayed extubation, early assessment of the need for continuous renal replacement therapy, and evaluation for mechanical circulatory support. Finally, when platelet counts fail to recover, clinicians should consider an evaluation for heparin-induced thrombocytopenia, including the 4Ts score and anti-PF4/heparin antibody testing, rather than attributing persistent thrombocytopenia solely to illness severity.

We developed a prediction model for early identification of patients at high risk for platelet count derangement postoperatively. The model incorporates five routinely collected perioperative parameters: age, preoperative creatinine, total transfusion volume, end-stage renal disease, and operation time. Because these variables are readily available from standard clinical documentation, the model can be implemented without additional testing, facilitating rapid integration into perioperative workflows. The visualized nomogram further supports individualized quantitative risk assessment, allowing clinicians to evaluate the risk of high-risk platelet trajectory preoperatively or in the early postoperative period to guide targeted intervention.

### Limitations

Several limitations of the present study need to be acknowledged when interpreting these results. First, the single-center retrospective design inherently introduces selection and information biases. Although we applied strict inclusion and exclusion criteria and used multiple imputation to address missing data, residual confounding could not be fully eliminated. Second, patients who died intraoperatively or within the first seven postoperative days were excluded to ensure complete longitudinal platelet records and reduce survival bias for GMM trajectory modeling. However, this exclusion removed critically ill patients who experienced early death, which may lead to underestimation of the true overall mortality risk among all patients with ATAAD. Third, this study only analyzed platelet counts, which cannot fully reflect actual platelet functional status. Moreover, routine screening for heparin-induced thrombocytopenia-associated antibodies was not available, and it was difficult to comprehensively trace all medications that might have affected platelet counts. Therefore, we cannot exclude the possibility that cases of HIT or drug-induced thrombocytopenia were included in Traje C. Fourth, model validation was performed only through internal random data splitting, without external verification using independent multicenter cohorts. Therefore, the external validity of this predictive model needs to be further validated in external prospective multicenter studies.

## Conclusion

In conclusion, we identified three distinct postoperative platelet-count trajectories in patients undergoing surgical repair of ATAAD using GMM. A persistently low platelet trajectory was independently associated with increased short- and long-term mortality. A convenient predictive model based on routine perioperative clinical indicators was further established for the early identification of high-risk platelet phenotypes. These findings challenge the conventional paradigm relying on static platelet measurements and highlight the unique prognostic value of longitudinal platelet trajectory analysis.

## Data Availability

The original contributions presented in the study are included in the article/Supplementary Material, further inquiries can be directed to the corresponding authors.
