# Supplementary material for: Postoperative platelet dynamic trajectories in patients with type A aortic dissection and development of a predictive model for thrombocytopenia
Source: Front Cardiovasc Med. 2026 Oct 7;13:1948231. doi: 10.3389/fcvm.2026.1948231 (PMC13683688; doi:10.3389/fcvm.2026.1948231)
Supplement: Supplementary file 1 [file Datasheet1.docx]

**Supplement**

**Table1 Analysis of differences in each characteristic between the training set and the validation set**

| **Variable** | **Set** | | **Statistic** | ***P*** |
| --- | --- | --- | --- | --- |
|  | **Train (n=542)** | **Test (n=231)** |  |  |
| Gender, n (%) |  |  | χ²=0.476 | 0.490 |
| female | 137 (25.28%) | 53 (22.94%) |  |  |
| male | 405 (74.72%) | 178 (77.06%) |  |  |
| Age, Mean±SD | 52.95±12.92 | 52.16±13.32 | t=0.772 | 0.441 |
| BMI, Mean±SD | 25.02±4.31 | 25.73±4.13 | t=0.772 | 0.503 |
| Hypertension, n (%) |  |  | χ²=0.257 | 0.612 |
| No | 148 (27.31%) | 59 (25.54%) |  |  |
| yes | 394 (72.69%) | 172 (74.46%) |  |  |
| Diabetes, n (%) |  |  | χ²=1.457 | 0.227 |
| No | 521 (96.13%) | 226 (97.84%) |  |  |
| yes | 21 (3.87%) | 5 (2.16%) |  |  |
| End Stage Renal Disease, n (%) |  |  | χ²=0.027 | 0.870 |
| No | 529 (97.60%) | 225 (97.40%) |  |  |
| yes | 13 (2.40%) | 6 (2.60%) |  |  |
| Smoke, n (%) |  |  | χ²=0.128 | 0.721 |
| No | 404 (74.54%) | 175 (75.76%) |  |  |
| yes | 138 (25.46%) | 56 (24.24%) |  |  |
| Alcohol, n (%) |  |  | χ²=0.131 | 0.718 |
| No | 447 (82.47%) | 188 (81.39%) |  |  |
| yes | 95 (17.53%) | 43 (18.61%) |  |  |
| Cerebralischemia preoperative, n (%) |  |  | χ²=0.325 | 0.569 |
| No | 495 (91.33%) | 208 (90.04%) |  |  |
| yes | 47 (8.67%) | 23 (9.96%) |  |  |
| Limbischemia preoperative, n (%) |  |  | χ²=0.362 | 0.548 |
| No | 471 (86.90%) | 197 (85.28%) |  |  |
| yes | 71 (13.10%) | 34 (14.72%) |  |  |
| Bowelischemia Preoperative, n (%) |  |  | χ²=1.488 | 0.223 |
| No | 522 (96.31%) | 218 (94.37%) |  |  |
| yes | 20 (3.69%) | 13 (5.63%) |  |  |
| Cororyartery involvement, n (%) |  |  | χ²=0.022 | 0.882 |
| No | 441 (81.37%) | 189 (81.82%) |  |  |
| yes | 101 (18.63%) | 42 (18.18%) |  |  |
| Preoperative hypotension, n (%) |  |  | χ²=1.802 | 0.180 |
| No | 502 (92.62%) | 220 (95.24%) |  |  |
| yes | 40 (7.38%) | 11 (4.76%) |  |  |
| Malperfusion syndrome, n (%) |  |  | χ²=0.182 | 0.670 |
| No | 321 (59.23%) | 133 (57.58%) |  |  |
| yes | 221 (40.77%) | 98 (42.42%) |  |  |
| Tampode, n (%) |  |  | χ²=0.624 | 0.430 |
| No | 485 (89.48%) | 211 (91.34%) |  |  |
| yes | 57 (10.52%) | 20 (8.66%) |  |  |
| Rootsurgery, n (%) |  |  | χ²=2.228 | 0.328 |
| Root Bentall | 111 (20.48%) | 42 (18.18%) |  |  |
| Root reconstruction | 416 (76.75%) | 186 (80.52%) |  |  |
| Root untreated | 15 (2.77%) | 3 (1.30%) |  |  |
| Archsurgery, n (%) |  |  | - | 0.292 |
| Hemiarch replacement | 91 (16.79%) | 43 (18.61%) |  |  |
| Total arch replacement with FET | 265 (48.89%) | 101 (43.72%) |  |  |
| Arch stent | 177 (32.66%) | 86 (37.23%) |  |  |
| Untreated | 9 (1.66%) | 1 (0.43%) |  |  |
| CABG, n (%) |  |  | χ²=0.099 | 0.754 |
| No | 510 (94.10%) | 216 (93.51%) |  |  |
| yes | 32 (5.90%) | 15 (6.49%) |  |  |
| Operation time hours, Mean±SD | 7.76±1.84 | 7.73±1.64 | t=0.226 | 0.821 |
| CPB min, Mean±SD | 232.70±65.60 | 238.21±63.85 | t=-1.076 | 0.282 |
| Aortic clamp min, Mean±SD | 163.32±52.61 | 166.56±50.90 | t=-0.791 | 0.429 |
| RBCs, Mean±SD | 6.31±4.47 | 6.23±4.06 | t=0.232 | 0.816 |
| Plasma, Mean±SD | 857.05±486.05 | 822.63±446.55 | t=0.923 | 0.356 |
| Cryo, Mean±SD | 11.03±6.03 | 11.05±4.97 | t=-0.039 | 0.969 |
| PC, Median (IQR) | 1.00 (1.00, 2.00) | 1.60 (1.00, 2.00) | Z=59828.500 | 0.289 |
| Total, Mean±SD | 2783.03±1463.82 | 2802.88±1346.82 | t=-0.177 | 0.860 |
| Preoperative platelet count, Mean±SD | 162.69±59.20 | 159.26±62.47 | t=0.725 | 0.468 |
| Preoperative WBC, Mean±SD | 12.11±4.08 | 12.30±4.20 | t=-0.604 | 0.546 |
| Preoperative HGB, Mean±SD | 129.61±21.86 | 130.22±20.65 | t=-0.357 | 0.721 |
| Preoperative PT, Median (IQR) | 12.20 (11.40, 13.40) | 12.20 (11.35, 13.47) | Z=63217.500 | 0.828 |
| Preoperative APTT, Median (IQR) | 28.40 (26.00, 33.84) | 29.10 (26.45, 34.90) | Z=59597.500 | 0.291 |
| Preoperative INR, Median (IQR) | 1.07 (0.99, 1.17) | 1.07 (0.99, 1.17) | Z=63206.500 | 0.831 |
| Preoperative AST, Median (IQR) | 33.38 (22.90, 69.27) | 32.00 (23.25, 65.45) | Z=63429.000 | 0.771 |
| Preoperative Cr, Median (IQR) | 81.65 (63.12, 111.00) | 78.70 (62.00, 111.00) | Z=63189.000 | 0.836 |
| Preoperative BUN, Median (IQR) | 6.76 (5.38, 8.48) | 6.61 (5.41, 8.20) | Z=63264.500 | 0.816 |

1. Mean±SD: Mean±standard deviation (n=sample size); Median: median; IQR: interquartile range; t: t test, if the homogeneity of variance was satisfied, the t-test was used for analysis; otherwise, the Welch’s t-test (t’-test) was applied.; Z: Wilcoxon rank-sum test; χ²: Chi-square test; -: Fisher's exact test.

2. Categorical variables were presented as number (percentage). For continuous variables, the mean, standard deviation and median were rounded to two decimal places, and the P value was rounded to three decimal places. Normally distributed continuous variables were expressed as mean±SD, while skewed variables were presented as median (IQR).

**Table2 Collinearity diagnosis results**

| **Variable** | **GVIF** | **Df** | **GVIF^(1/(2*Df))** |
| --- | --- | --- | --- |
| CPB min | 3.51 | 1 | 1.87 |
| Aortic clamp min | 3.39 | 1 | 1.84 |
| Preoperative_Cr | 2.16 | 1 | 1.47 |
| Operation timehours | 1.91 | 1 | 1.38 |
| Smoke | 1.82 | 1 | 1.35 |
| Preoperative HGB | 1.79 | 1 | 1.34 |
| Preoperative BUN | 1.77 | 1 | 1.33 |
| Alcohol | 1.74 | 1 | 1.32 |
| Gender | 1.51 | 1 | 1.23 |
| End Stage Renal Disease | 1.50 | 1 | 1.22 |
| Age | 1.41 | 1 | 1.19 |
| Root surgery | 1.89 | 2 | 1.17 |
| total | 1.38 | 1 | 1.17 |
| Pre operative WBC | 1.33 | 1 | 1.15 |
| BMI | 1.31 | 1 | 1.14 |
| Arch surgery | 2.04 | 3 | 1.13 |
| CABG | 1.25 | 1 | 1.12 |
| Cororyartery involvement | 1.23 | 1 | 1.11 |
| Tampode | 1.23 | 1 | 1.11 |
| Preoperative AST | 1.24 | 1 | 1.11 |
| Hypertension | 1.18 | 1 | 1.09 |
| Preoperative hypotension | 1.16 | 1 | 1.08 |
| Preoperative INR | 1.14 | 1 | 1.07 |
| Preoperative platelet count | 1.13 | 1 | 1.06 |
| Limbischemia preoperative | 1.10 | 1 | 1.05 |
| Cerebralischemia preoperative | 1.09 | 1 | 1.04 |
| Bowelischemia preoperative | 1.06 | 1 | 1.03 |
| Diabetes | 1.04 | 1 | 1.02 |

**Table3 Bootstrap frequency table**

| **Variable** | **Count** | **Percentage** |
| --- | --- | --- |
| Age | 1,000 | 100.00 |
| Preoperative_Cr | 991 | 99.10 |
| total | 937 | 93.70 |
| End Stage Renal Disease | 803 | 80.30 |
| Operation time hours | 726 | 72.60 |
| CPB min | 592 | 59.20 |
| Preoperative hypotension | 577 | 57.70 |
| Cororyartery involvement | 567 | 56.70 |
| Limbischemia preoperative | 558 | 55.80 |
| Arch surgery | 544 | 54.40 |
| Preoperative platelet count | 508 | 50.80 |
| Preoperative AST | 503 | 50.30 |
| Tampode | 472 | 47.20 |
| Diabetes | 421 | 42.10 |
| Preoperative_HGB | 390 | 39.00 |
| Gender | 328 | 32.80 |
| Hypertension | 307 | 30.70 |
| Cerebralischemia preoperative | 282 | 28.20 |
| Smoke | 238 | 23.80 |
| CABG | 238 | 23.80 |
| Bowelischemia preoperative | 202 | 20.20 |
| Preoperative INR | 118 | 11.80 |
| Preoperative BUN | 115 | 11.50 |
| Preoperative WBC | 102 | 10.20 |
| Root surgery | 100 | 10.00 |
| Alcohol | 96 | 9.60 |
| BMI | 94 | 9.40 |
| Aortic clamp min | 53 | 5.30 |

**Table4 Bootstrap frequency table**

| Variable | GVIF | Df | GVIF^(1/(2*Df)) |
| --- | --- | --- | --- |
| Preoperative INR | 47.81 | 1 | 6.91 |
| Preoperative PT | 47.78 | 1 | 6.91 |
| total | 24.59 | 1 | 4.96 |
| RBCs | 10.52 | 1 | 3.24 |
| Malperfusion syndrome | 5.31 | 1 | 2.30 |
| plasma | 4.52 | 1 | 2.13 |
| CPB min | 3.58 | 1 | 1.89 |
| Aorticclamp min | 3.48 | 1 | 1.86 |
| Cororyartery involvement | 3.02 | 1 | 1.74 |
| Preoperative_Cr | 2.18 | 1 | 1.48 |
| PC | 2.16 | 1 | 1.47 |
| Limbischemia preoperative | 1.95 | 1 | 1.40 |
| Cerebralischemia preoperative | 1.94 | 1 | 1.39 |
| Operation time hours | 1.92 | 1 | 1.38 |
| Smoke | 1.86 | 1 | 1.36 |
| Preoperative_HGB | 1.85 | 1 | 1.36 |
| Cryo | 1.83 | 1 | 1.35 |
| Alcohol | 1.78 | 1 | 1.33 |
| Preoperative BUN | 1.77 | 1 | 1.33 |
| End Stage Renal Disease | 1.54 | 1 | 1.24 |
| Gender | 1.53 | 1 | 1.24 |
| Age | 1.43 | 1 | 1.20 |
| Root surgery | 1.93 | 2 | 1.18 |
| Preoperative hypotension | 1.40 | 1 | 1.18 |
| Bowelischemia preoperative | 1.35 | 1 | 1.16 |
| Preoperative WBC | 1.34 | 1 | 1.16 |
| BMI | 1.33 | 1 | 1.15 |
| Preoperative APTT | 1.32 | 1 | 1.15 |
| Arch surgery | 2.13 | 3 | 1.13 |
| Preoperative AST | 1.27 | 1 | 1.13 |
| CABG | 1.26 | 1 | 1.12 |
| Tampode | 1.25 | 1 | 1.12 |
| Hypertension | 1.20 | 1 | 1.09 |
| Preoperative platelet count | 1.16 | 1 | 1.08 |
| Diabetes | 1.05 | 1 | 1.02 |

**Figure 1 ROC curve plot**

This ROC curve was re-plotted using the total intraoperative transfusion volume after subtracting the volume of intraoperative platelet transfusion. The ROC curve showed an AUC of 0.73, consistent with the C statistic of 0.73 in the calibration plot, indicating that the model has moderate-to-good discriminative ability to effectively distinguish patients with different risk levels.

**Figure 2 Risk prediction nomogram based on logistic regression prediction model**

This nomogram was re-plotted using the total intraoperative transfusion volume after subtracting the volume of intraoperative platelet transfusion.(Total_plate=total intraoperative transfusion volume after subtracting the volume of intraoperative platelet transfusion)

**Figure 3 Decision Curve Analysis (DCA) for the logistic regression model**

This DCA was re-plotted using the total intraoperative transfusion volume after subtracting the volume of intraoperative platelet transfusion. DCA demonstrated that the model provided a greater net benefit than the treat-all and treat-none strategies across most of the threshold probability range (approximately 0.05–0.90), supporting its clinical utility.

**Figure 4 Calibration curves**

This Calibration curves was re-plotted using the total intraoperative transfusion volume after subtracting the volume of intraoperative platelet transfusion. The Hosmer–Lemeshow test yielded a chi-squared value of 10.695 (P = 0.22). A P value greater than 0.05 indicates no significant difference between the predicted probabilities and the observed outcomes, suggesting good calibration of the model.
